# Recombinant IL1-RA in the early phase of systemic onset JIA: before the onset of arthritis

**DOI:** 10.1186/1546-0096-9-S1-O6

**Published:** 2011-09-14

**Authors:** SJ Vastert, W De Jager, BJ Noordman, D Holzinger, W Kuis, BJ Prakken, NM Wulffraat

**Affiliations:** 1 Pediatric Immunology, University Medical Center Utrecht, The Netherlands; 2 Institute of Immunology, University Hospital Münster, Münster, Germany

## Background

Systemic Onset Juvenile Idiopathic Arthritis (SoJIA) is characterized by systemic inflammation besides arthritis. SoJIA has a broad differential diagnosis. In a minority of SoJIA patients, it takes time (weeks to months) before arthritis develops. These cases are challenging for clinicians, as is the question how and when to treat these patients.

## Aim

to identify SoJIA patients before the onset of arthritis and to evaluate treatment with recombinant IL-1RA (Anakinra) in these ‘suspected SoJIA’ patients.

## Methods

We characterized the systemic inflammation in 5 Â´suspected SoJIAÂ´ patients and compared them to our cohort of known SoJIA patients. These 5 patients were thoroughly checked for alternative diagnoses by extensive microbial analysis (PCR, cultures), radiological evaluation (including CT and/or PET scans) and genetic testing where appropriate. Moreover, bonemarrow aspirates were performed to rule out leukemia and haemophagocytosis.

Biochemical and immunological parameters of disease activity (ESR, CRP, Ferritin) as well as newly developed biomarkers for SoJIA (cytokine profiles (IL-18), MRPÂ´s, NK cell numbers, phenotype and lytic function) were assessed at onset of disease and during treatment with Anakinra.

## Results

The 5 ‘suspected SoJIA’ patients were clinically and immunologically very similar to our cohort of SoJIA patients (table 1) at time of their diagnosis. We started Anakinra (dose 2mg/kg/day) in these patients and prospectively followed them for 10 months (5-12) months. All 5 patients showed beneficial responses to Anakinra, with fast resolution of inflammation and improved NK cell function.

**Table 1:** 

Disease mark	Suspected SoJIA patients (without arthritis, n=5)	SoJIA cohort Utrecht (n=15)
Spiking fever	5/5	15/15
Exanthema	2/5	12/15
Arthritis	0/5	15/15
CRP (mg/L)	112±13	209±18
BSE (mm/hr)	128±26	127±34
Ferritin (ug/L)	937±1118	2405±3855
MRP8/14 (U/L)	38490±23077	45185±37260
IL-18 plasma (ng/L)	3.6±1.7	8.2±9.0
Abs. NK cell (x10^9^/L)	0.096±0.114	0.092±0.132
NK cell function (% killing)	4.9±3.7	6.5±4.5

## Conclusion

Here we show that, making use of recently developed biomarkers, `suspected SoJIAÂ´ patients can be identified very early in their disease-course, before the development of arthritis. These patients show a similar beneficial clinical response to treatment with recombinant IL-1RA as our cohort of newly diagnosed SoJIA patients. Our data support the idea of early treatment with recombinant IL-1RA in (suspected) SoJIA patients.

